# Pneumococcal Conjugate Vaccine Impact on Meningitis and Pneumonia Among Children Aged <5 Years—Zimbabwe, 2010–2016

**DOI:** 10.1093/cid/ciz462

**Published:** 2019-09-05

**Authors:** Vongai Dondo, Hilda Mujuru, Kusum Nathoo, Vengai Jacha, Ottias Tapfumanei, Priscilla Chirisa, Portia Manangazira, John Macharaga, Linda de Gouveia, Jason M Mwenda, Regis Katsande, Goitom Weldegebriel, Tracy Pondo, Almea Matanock, Fernanda C Lessa

**Affiliations:** 1 Department of Paediatrics and Child Health, University of Zimbabwe, Harare, Zimbabwe; 2 Harare Central Hospital, Harare, Zimbabwe; 3 Epidemiology and Disease Control, Ministry of Health and Child Care, Harare, Zimbabwe; 4 Centre for Respiratory Diseases and Meningitis, National Institute for Communicable Diseases of the National Health Laboratory Service, Johannesburg, South Africa; 5 World Health Organization, Regional Office for Africa, Brazzaville, Republic of Congo; 6 World Health Organization, Intercountry Support Team, Harare, Zimbabwe; 7 Respiratory Diseases Branch, Centers for Disease Control and Prevention, Atlanta, Georgia

**Keywords:** pneumococcal vaccines, pneumonia, meningitis, children, Zimbabwe

## Abstract

**Background:**

*Streptococcus pneumoniae* is a leading cause of pneumonia and meningitis in children aged <5 years. Zimbabwe introduced 13-valent pneumococcal conjugate vaccine (PCV13) in 2012 using a 3-dose infant schedule with no booster dose or catch-up campaign. We evaluated the impact of PCV13 on pediatric pneumonia and meningitis.

**Methods:**

We examined annual changes in the proportion of hospitalizations due to pneumonia and meningitis among children aged <5 years at Harare Central Hospital (HCH) pre-PCV13 (January 2010–June 2012) and post-PCV13 (July 2013–December 2016) using a negative binomial regression model, adjusting for seasonality. We also evaluated post-PCV13 changes in serotype distribution among children with confirmed pneumococcal meningitis at HCH and acute respiratory infection (ARI) trends using Ministry of Health outpatient data.

**Results:**

Pneumonia hospitalizations among children aged <5 years steadily declined pre-PCV13; no significant change in annual decline was observed post-PCV13. Post-PCV13 introduction, meningitis hospitalization decreased 30% annually (95% confidence interval [CI], –42, –14) among children aged 12–59 months, and no change was observed among children aged 0–11 months. Pneumococcal meningitis caused by PCV13 serotypes decreased from 100% in 2011 to 50% in 2016. Annual severe and moderate outpatient ARI decreased by 30% (95% CI, –33, –26) and 7% (95% CI, –11, –2), respectively, post-PCV13 introduction.

**Conclusions:**

We observed declines in pediatric meningitis hospitalizations, PCV13-type pneumococcal meningitis, and severe and moderate ARI outpatient visits post-PCV13 introduction. Low specificity of discharge codes, changes in referral patterns, and improvements in human immunodeficiency virus care may have contributed to the lack of additional declines in pneumonia hospitalizations post-PCV13 introduction.

## Teaser

In 2012, Zimbabwe introduced 13-valent pneumococcal conjugate vaccine (PCV13) into the routine pediatric vaccination program. After its introduction, we observed declines in pediatric meningitis hospitalizations, PCV13-type pneumococcal meningitis, and outpatient visits for severe and moderate acute respiratory infections.

Pneumonia is a leading cause of mortality among children aged <5 years worldwide, accounting for 16% of 5.6 million deaths in this age group in 2016 [1]. The largest proportion of the disease burden is in resource-limited countries. In Zimbabwe, pneumonia is the leading cause of childhood deaths outside of the neonatal period [1]. There are many causes of pneumonia, which are difficult to identify and accurately measure [2, 3], but *Streptococcus pneumoniae* is well established as a leading bacterial cause [4]. In addition to pneumonia, *S. pneumoniae* can also cause invasive pneumococcal disease (IPD), which includes sepsis and meningitis. The relatively large burden of human immunodeficiency virus (HIV; 15% prevalence in 2010) in Zimbabwe increases the risk of IPD [5]. Before antiretroviral therapy was widely used, HIV imparted a 42-fold increased risk of IPD among children aged <5 years [6]. Malnutrition and tuberculosis further increase the risk of IPD in children with HIV infection compared to those without HIV infection [7].

In 2007, the World Health Organization (WHO) recommended that pneumococcal conjugate vaccine (PCV) be included in routine pediatric immunization schedules worldwide [8]. In recent years, the majority of countries in Africa have introduced PCV. With support from Gavi, the Vaccine Alliance, Zimbabwe introduced 13-valent pneumococcal conjugate vaccine (PCV13) nationwide in July 2012, using a 3-dose primary series schedule at 6, 10, and 14 weeks of age. PCV13 coverage for all 3 doses has stayed above 85% since 2013 [9].

Few studies have attempted to evaluate the impact of PCV on IPD or pneumonia in resource-limited settings where pneumococcal disease surveillance is not routine [10–12]. Without routine surveillance for pneumococcal disease, other data sources and study designs are needed to fill this information gap. A recent study in Rwanda used admission logs to evaluate PCV impact [11]. Evaluation of population-level PCV impact using administrative datasets has been common in developed countries [13, 14]. However, the use of administrative data in Africa to evaluate PCV impact has been limited.

We used hospital discharge data from a large referral pediatric hospital in Zimbabwe to evaluate the impact of PCV13 introduction on all-cause pneumonia and meningitis among children aged <5 years. Using data from the same hospital, we also examined the proportion of pneumococcal meningitis caused by PCV13 serotypes over time. Finally, we used national-level outpatient data from the Ministry of Health to evaluate changes in the frequency of acute respiratory illness (ARI) visits post-PCV13 introduction. We examined trends over time (as opposed to pre–post percent change) to evaluate for other potential unmeasured interventions and changes in referral patterns that may have also affected pneumonia and meningitis hospitalization rates.

## METHODS

### Surveillance for Pneumonia and Meningitis at a Sentinel Site Hospital

Harare Central Hospital (HCH) is the largest public pediatric hospital in Zimbabwe, with 345 pediatric beds. Although patients from anywhere in the country can be admitted to HCH, the hospital serves mainly the greater Harare area, which has a catchment population of 2.1 million. We used the hospital discharge database to identify all-cause pneumonia, bronchiolitis (to assess potential misclassification of pneumonia hospitalizations), and meningitis hospitalizations among children aged 0–11 months and 12–59 months from 1 January 2010 to 31 December 2016 based on discharge codes (Supplementary Materials). HCH transitioned from *International Classification of Diseases, Ninth Revision, Clinical Modificatio*n (ICD9) to *International Classification of Diseases, Tenth Revision, Clinical Modificatio*n (ICD10) in 2014 [15].

### Active Surveillance for Suspected Meningitis

HCH is a sentinel site for the WHO’s Invasive Bacterial-Vaccine Preventable Disease Surveillance network. As part of this surveillance, children aged <5 years who meet the case definition for suspected meningitis are enrolled. A suspected case of meningitis is defined as illness in a child aged <5 years admitted with sudden onset of fever (>38.5°C rectal or 38.0°C axillary) and 1 of the following signs: neck stiffness, bulging fontanel, altered consciousness, convulsion, or other meningeal sign or a clinical diagnosis of meningitis. Cerebrospinal fluid (CSF) was collected from all children with suspected meningitis, where caregiver consent was given, and sent to the hospital laboratory for processing, including biochemistry, cell count, culture, and, when available, antigen test (BinaxNOW) or bacterial latex agglutination for *S. pneumoniae*. Starting in 2011, if sufficient volume (0.5 mL) remained after laboratory processing, the residual CSF sample was sent to the South African National Institute for Communicable Diseases (NICD) for detection of *S. pneumoniae* by real-time polymerase chain reaction (PCR). All CSF specimens that were positive by PCR for *S. pneumoniae* underwent multiplex PCR for serotyping [16]. When available, pneumococcal isolates were also sent to NICD for confirmation and serotyping by Quellung reaction.

### Nationwide Outpatient Surveillance for Respiratory Infections

The Health Information and Surveillance Unit of the Department of Epidemiology and Disease Control of the Ministry of Health and Child Care receives monthly reports from 1850 health institutions on the District Health Information System (DHIS) 2 platform. Monthly DHIS2 reports include number of children seen for mild, moderate, and severe ARI. Mild ARI was defined as acute onset of cough, congestion, or sore throat without signs of moderate or severe pneumonia. Moderate ARI was defined as lower respiratory infection with fast breathing or chest in-drawing but without signs of severe pneumonia. Severe ARI was defined as lower respiratory infection and at least 1 of the following: central cyanosis or oxygen saturation <90%, severe respiratory distress, inability to drink, lethargy, loss of consciousness, or convulsions. Because DHIS2 was implemented in 2012, we examined the number of outpatient visits for mild, moderate, and severe ARI among children aged <5 years from January 2012 through December 2016. We limited our analysis to health facilities with ≥200 severe ARI visits per year and ≥1 visit in every month of the year in order to be able to assess trends. Additionally, we excluded 2 facilities that had an unexplained increase of >500 mild and moderate ARI visits in a single month.

### Statistical Analyses

We defined the pre-PCV13 introduction period as January 2010–June 2012 and the post-PCV13 introduction period as July 2013–December 2016. The first year of PCV13 introduction (July 2012–June 2013) was excluded to allow for vaccine uptake. We used a negative binomial segmented regression model to calculate the annual percent change in each outcome of interest (pneumonia, bronchiolitis, and meningitis) by age group (0–11 months and 12–59 months) during the pre- and post-PCV13 introduction periods. Using the model, we examined the change in slope pre- and post-PCV13 introduction to determine if there was a statistically significant (*P* ≤ .05) change in the time period after vaccine introduction. In the model, the dependent variable was the number of hospitalizations for a particular diagnosis. Initially, we sought to compare our outcomes of interest to a control condition. However, after excluding congenital diseases, 80% of hospitalizations were for infectious causes or complications of infectious diseases such as dehydration and acute respiratory failure. The remaining 20% of hospitalizations for noninfectious causes were susceptible to large month-to-month fluctuations. Therefore, we decided to use the total number of hospitalizations, which were relatively stable over time, as the offset variable (the variable used to generate the rate in the regression model). Additionally, we included harmonic terms to account for seasonality. For outpatient visits for mild, moderate, and severe ARI, we did not have a comparison pre-PCV13 introduction period nor an offset variable, so we analyzed the annual percent decline using monthly counts in the post-PCV13 period. Data were analyzed using SAS version 9.4 (SAS Institute Inc., Cary, NC) and Microsoft Office Excel version 2016.

This project was reviewed in accordance with the Centers for Disease Control and Prevention and WHO human research protection procedures and was determined to be nonresearch. It was considered part of national public health surveillance in Zimbabwe, and so it did not require local institutional review board review.

## RESULTS

### Surveillance for Pneumonia and Meningitis at a Sentinel Site Hospital

Among children aged 0–11 months, there was an average of 904 children total all-cause hospitalized per month pre-PCV13 introduction and 859 post-PCV13 introduction (Table 1). Among children aged 12–59 months, there was an average of 433 children total all-cause hospitalized per month pre-PCV13 introduction and 465 post-PCV13 introduction. The average monthly number of admissions for pneumonia and meningitis declined pre- to post-PCV13 introduction, as did the case fatality ratio (CFR) for both syndromes, with the exception of the meningitis CFR for children aged 12–59 months, which was unchanged (21% pre-PCV13 vs 20% post-PCV13 introduction).

**Table 1.** Hospitalizations for Pneumonia and Meningitis Pre– and Post–13-Valent Pneumococcal Conjugate Vaccine Introduction, per Month, 2010–2016

Age Group, months	Time Period	Pneumonia, n (%)	Pneumonia CFR, %	Meningitis, n (%)	Meningitis, CFR, %	Total Hospitalizations
0–11	Pre-PCV13 (January 2010–June 2012)	145 (16)	12	9 (1)	25	904
	Post-PCV13 (July 2013–December 2016)	102 (12)	8	8 (0.5)	16	859
12–59	Pre-PCV13 (January 2010–June 2012)	81 (19)	7	5 (1)	21	433
	Post-PCV13 (July 2013–December 2016)	66 (14)	4	2 (0.5)	20	465

Abbreviations: CFR, case fatality ratio; PCV13, 13-valent pneumococcal conjugate vaccine.

#### Pneumonia

**Figure 1.**
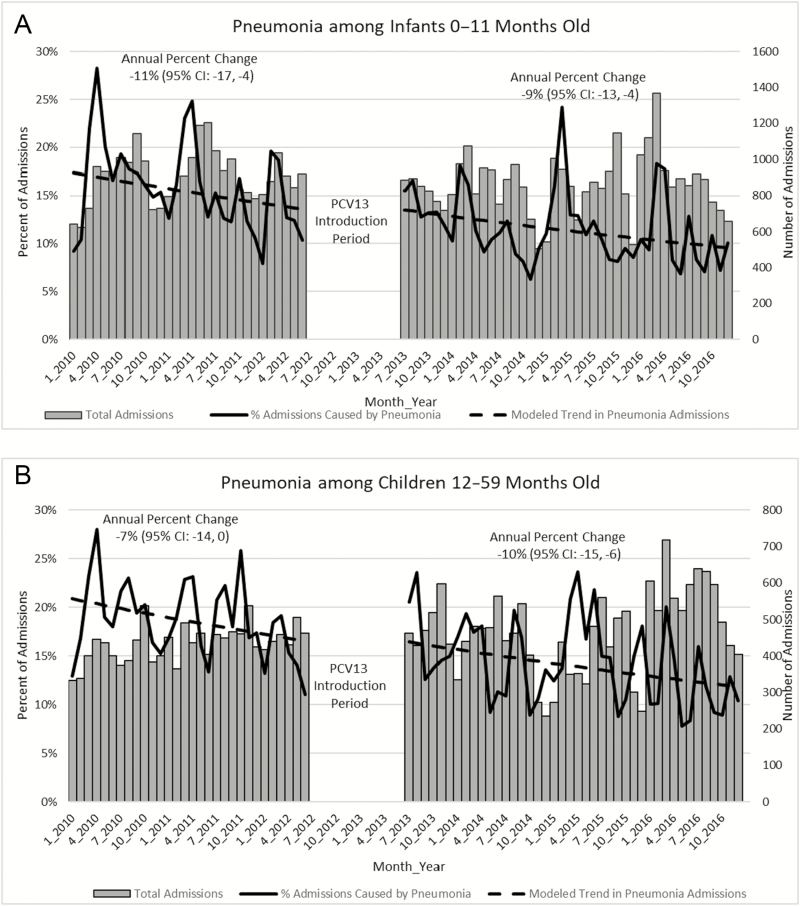

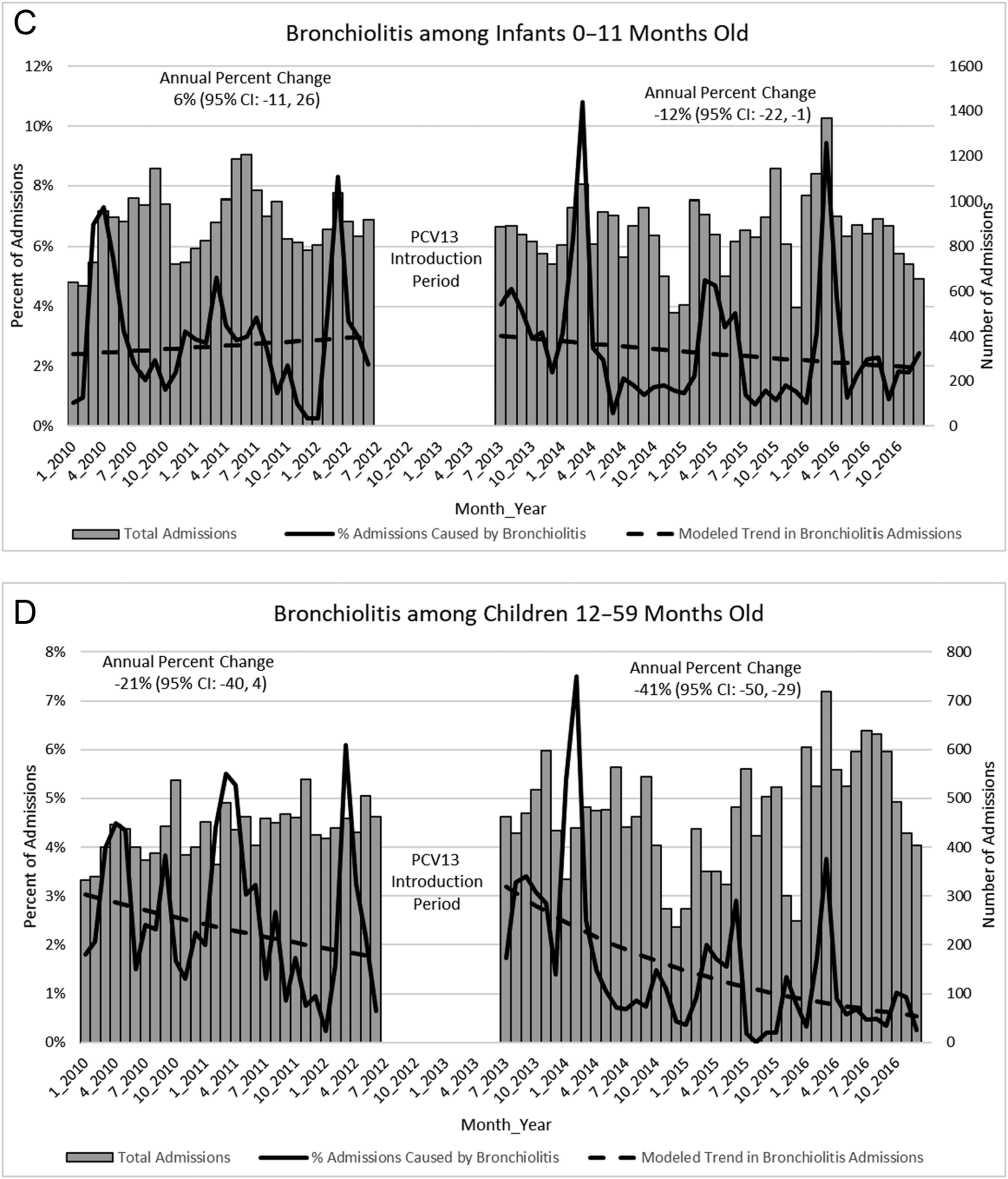

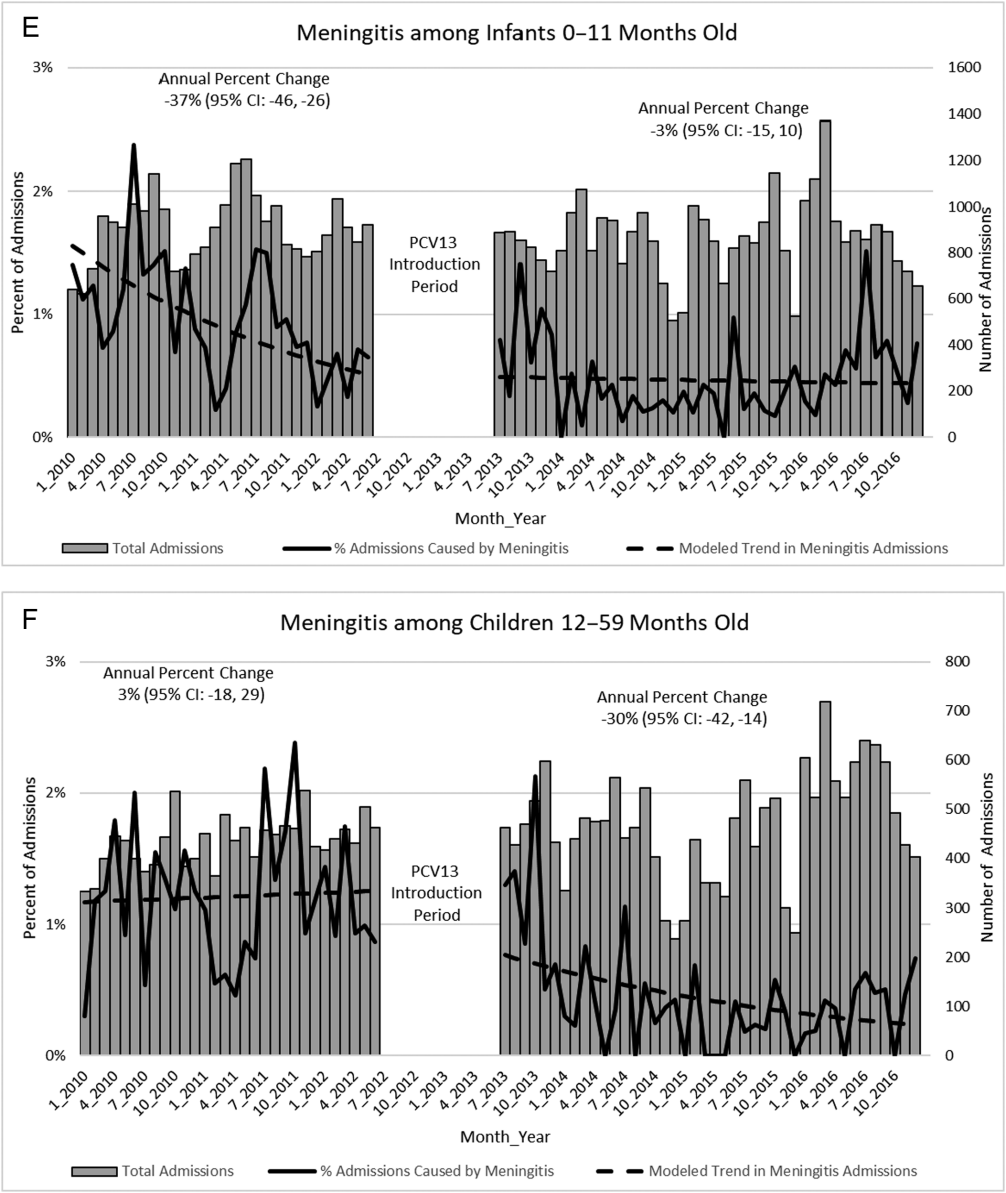
Harare Central Hospital admissions by syndrome and age group, 2010–2016. Abbreviations: CI, confidence interval; PCV13, 13-valent pneumococcal conjugate vaccine.

Declines in pneumonia hospitalizations among children aged 0–11 months were observed pre-PCV13 introduction (annual percent change, –11%; 95% confidence interval [CI], –17, –4) and continued post-PCV13 introduction (annual percent change, –9%; 95% CI, –13, –4]) without a statistically significant change between pre- and post-PCV13 introduction (*P* = .7; Figure 1). Pneumonia hospitalizations among children aged 12–59 months declined pre-PCV13 introduction, although it was not a statistically significant decline (–7%; 95% CI, –14, 0). A significant annual decline post-PCV13 introduction was observed (–10%; 95% CI, –15, –6). Despite the declines during the post-PCV13 period, there was no significant difference between the trends of pneumonia hospitalization among children aged 12–59 months pre- and post-PCV13 introduction (*P* = .6). For comparison, we also looked at the trend in bronchiolitis pre- and post-PCV13 introduction (Figure 1). Similar to pneumonia, a nonsignificant decline in bronchiolitis hospitalization was observed among children aged 0–11 and 12–59 months pre-PCV13. However, significant annual declines in bronchiolitis hospitalizations of 12% (95% CI, –22, –1) among children aged 0–11 months and 41% (95% CI, –50, –29) among children aged 12–59 months were observed post-PCV13. Despite the declines in the post-PCV13 period, there was no change in the trends pre- to post-PCV13 (*P* = .2 for children aged 0–11 months and *P* = .08 for children aged 12–59 months).

Seasonal peaks in pneumonia hospitalizations occurred between January and March. Pre-PCV13, 25% of all hospitalizations were due to pneumonia during the highest seasonal peaks. This decreased to 20% post-PCV13 (Figure 1). We did not observe a similar seasonal effect for bronchiolitis.

#### Meningitis

Declines in meningitis hospitalizations among children aged 0–11 months were observed primarily in the pre-PCV13 period (annual percent change –37%; 95% CI, –46, –26; Figure 1). The declines continued until the first year after PCV13 introduction and then plateaued in the following years. Among children aged 12–59 months, there was a statistically significant decrease in meningitis hospitalizations post-PCV13 introduction (annual percent change, –30%; 95% CI, –42, –14) compared to pre-PCV13 introduction (*P* = .02). Neonatal meningitis could not be analyzed separately.

**Figure 2.**
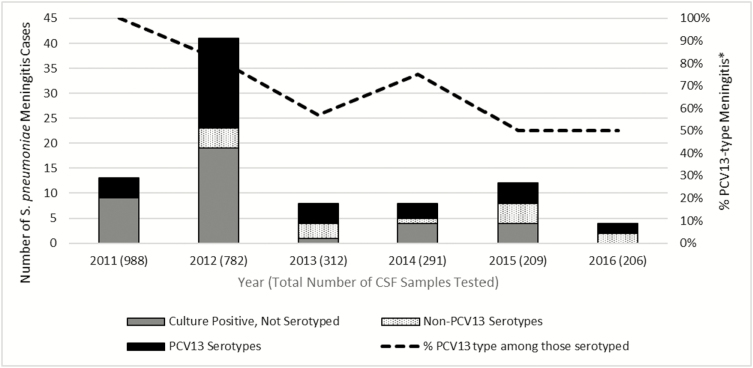
*Streptococcus pneumoniae*–positive CSF samples by serotype group among children aged <5 years admitted to Harare Central Hospital, 2011–2016. Abbreviations: CSF; cerebrospinal fluid; PCV13, 13-valent pneumococcal conjugate vaccine. *Calculated as the percent of PCV13-type among positive pneumococcal CSF specimens serotyped.

From 2011 through 2016, between 206 and 988 CSF samples were tested for *S. pneumoniae* per year among children aged <5 years. Overall, 1%–5% of samples tested positive for *S. pneumoniae* by PCR, culture, antigen test, or agglutination test per year. The proportion of pneumococcal meningitis that was caused by PCV13 serotypes decreased from 100% in 2011 to 50% in 2016 (Figure 2). The proportion of pneumococcal-positive CSF specimens for which serotype was not available also decreased over time. Data on PCV13 vaccination status for individual patients was not captured as part of the surveillance and therefore cannot be determined for the children with pneumococcal meningitis.

### Nationwide Outpatient Surveillance for Respiratory Infections

**Figure 3.**
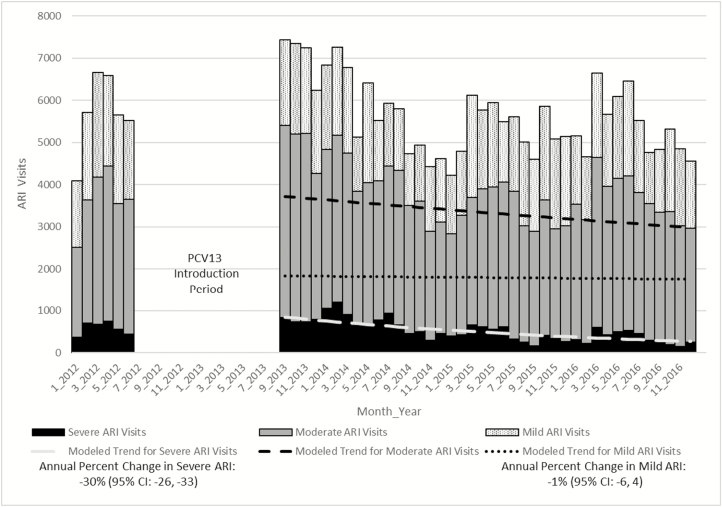
Nationwide ARI outpatient department visits among children aged <5 years, 2012–2016. Abbreviations: ARI, acute respiratory infection; PCV13, 13-valent pneumococcal conjugate vaccine.

We excluded 1835 facilities that had fewer than 200 severe ARI visits per year, 5 facilities with fewer than 1 visit in every month, and an additional 2 facilities where there was an unexplained increase in monthly ARI visits by more than 500 visits in a single month. After these exclusions, 8 outpatient clinics met our inclusion criteria. Severe and moderate ARI visits decreased post-PCV13 introduction (annual percent change in severe ARI was –30%; 95% CI, –33, –26 and in moderate ARI it was –7%; 95% CI, –11, –2; Figure 3). Seasonal peaks, seen best among the severe ARI case counts, were not as pronounced as peaks among inpatient pneumonia hospitalizations at HCH and occurred later in the year (May–August).

## DISCUSSION

Following PCV13 introduction, we observed a significant decrease in meningitis hospitalizations among children aged 12–59 months at the largest referral pediatric hospital in Zimbabwe. There was a 37% reduction in meningitis hospitalizations among children aged 0–11 months pre-PCV13 introduction, with no change post-PCV13 introduction. A decline in the proportion of PCV13 serotypes among isolates obtained from patients with pneumococcal meningitis was also observed. In previous studies, PCV introduction was associated with a 55%–56% decrease in incidence of IPD, including meningitis, among children aged 2–59 months [17] and with a 41%–49% decrease in incidence of pneumococcal meningitis among children aged 0–59 months [18].

Although pneumonia hospitalizations steadily declined over the entire study period, no significant changes post-PCV13 introduction were observed at HCH. This finding is similar to a recent finding from Rwanda in which no declines in pneumonia hospitalizations were observed at a single referral hospital following PCV introduction [11]. However, when a larger and more representative sample of district hospitals was included, the investigators were able to estimate a 54% (95% CI, 42, 63%) vaccine effectiveness against severe pneumonia among children aged <5 years using an indirect cohort method, suggesting that analyses with administrative data are more robust when multiple hospitals are included. It is also possible that PCV impact on pneumonia will cause more gradual declines over time at a single large referral hospital.

HCH is a referral hospital that receives critically ill children from district and provincial hospitals. Therefore, trends in disease at HCH may not be reflective of national trends but rather reflect the hospital’s capacity to care for critically ill children referred from district and provincial hospitals. The PCV13 coverage rates for Harare for the period 2013–2016 ranged from 84% to 104%, while national coverage rates were lower at 87% to 92%. Haemophilus *influenzae* type b (Hib) vaccine, introduced in 2008, may have further reduced pneumonia hospitalizations prior to the study period, 2010–2016. Hib vaccine coverage during the study period ranged from 84% to 106% in Harare and 82% to 102% nationally.

We did not see a decrease in total hospitalizations at HCH despite improvements in HIV care during the same period [5], rotavirus vaccine introduction in 2014 [19], and PCV13 introduction, as would have been expected. During the study period, there were simultaneous increases at HCH in admissions for other notable conditions such as malnutrition, leading to a relatively stable number of total hospitalizations. Additionally, other respiratory pathogens such as respiratory syncytial virus (RSV), influenza, parainfluenza, adenovirus, and *Mycoplasma pneumoniae*, to name a few, can also cause pneumonia in young children. All of these pathogens have the potential to cause outbreaks and most have a peak season that overlaps with that of pneumococci. For example, RSV outbreaks have been documented between February and June. These other pathogens may obscure our ability to detect the impact of PCV13 introduction on pneumonia. We did observe a decrease in the CFR for children admitted with pneumonia and a decrease in the seasonal peaks for pneumonia hospitalizations. These measures, along with the downward trend in pneumonia hospitalizations, indicate improvements in the prevention of pneumonia in children and the care of pediatric patients with pneumonia. However, we could not determine the impact of PCV13 introduction alone on pneumonia hospitalizations.

Lack of specificity of discharge codes for our outcomes of interest may have influenced our results. Pneumonia was defined by discharge codes alone (Supplementary Materials), which limited our ability to assess the severity and pathogen-specific etiologies of these respiratory infections. A recent study in which the impact of PCV10 among Kenyan children was evaluated demonstrated double the reduction in radiographically confirmed pneumonia that was seen in only clinically diagnosed pneumonia [20].

Additionally, changes in coding practices may have influenced our results. A relatively small number of codes are used in Zimbabwe (Supplementary Materials). However, the country switched from ICD9 to ICD10 in 2014, which may have affected our trends over time. As shown in the United States, transition from one version of ICD code to another can affect syndrome-specific hospitalization trends [15]. Furthermore, by using a pneumonia definition based on ICD codes, we may have missed some cases and misclassified others. For this reason, we evaluated the trends in bronchiolitis. We did not observe an increase in bronchiolitis post-PCV13, suggesting that the downward trends observed in pneumonia post-PCV13, even though not significant, were not related to misclassification of pneumonia into bronchiolitis.

After vaccine introduction, we observed a decrease in outpatient visits for moderate and severe ARI. We did not observe a decrease in visits for mild ARI, a finding consistent with that of other studies, likely because *S. pneumoniae* causes more severe ARI [11, 12]. These decreases were observed even after revised recommendations for management of pediatric pneumonia, including management of pneumonia with fast breathing in the outpatient clinic, were published in the 2014 WHO’s Integrated Management of Childhood Illness [21]. However, it is possible that Zimbabwe has not fully implemented the changes in pneumonia case management and, as a result, children with pneumonia who have fast breathing or chest in-drawing are still being hospitalized for treatment.

Our results are likely influenced by improvements in HIV prevention, care, and treatment during the period of this evaluation. From 2010 through 2016, the number of children living with HIV decreased from 110 000 to 81 000 [5]. A large part of this decline was driven by programs for the prevention of maternal-to-child transmission of HIV, which have expanded to 94% of coverage of pregnant women in 2016 vs 39% in 2010 [5, 22]. Pediatric HIV care and treatment in Zimbabwe has also improved, with early infant diagnosis increasing from 12% in 2010 to 71% in 2016 [5]. Early diagnosis can lead to earlier initiation of highly active antiretroviral treatment (HAART). Studies in Malawi, Mozambique, and South Africa, countries that also have a high prevalence of HIV, demonstrated declines in IPD after introduction of HAART among PCV-naive children [23–25]. The declines in IPD observed with HAART are similar to the declines seen in IPD post-PCV introduction.

Our evaluation has several limitations. First, we included only 1 referral hospital and a limited number of outpatient departments in our analysis. Therefore, our results are not generalizable to all of Zimbabwe. Second, we were unable to find suitable control conditions. Therefore, our analysis is subject to confounders inherent to hospitalization data, such as changes in healthcare utilization and coding practices. Third, active surveillance for bacterial meningitis with referral of specimens for molecular testing did not start at HCH until 2011; only one and a half years of data were available from pre-PCV13 introduction. Fourth, we were unable to stratify our data further by age. If we could have, we would have examined neonatal meningitis separately since it is less commonly caused by *S. pneumoniae* [26].

Despite these limitations, we were able to demonstrate declines in meningitis hospitalizations among children aged 12–59 months post-PCV13 introduction. We also documented declines in the percentage of pneumococcal meningitis caused by PCV13 serotypes using active surveillance data. Due to nonspecificity of all-cause pneumonia diagnosis, especially as measured in discharge codes, we were unable to discern the specific impact of PCV13 introduction. Improved access to routine clinical diagnostic tests would further advance our understanding of respiratory diseases in Zimbabwe. Although we were unable to observe an impact of PCV13 on pneumonia hospitalizations at a single referral hospital, declines in ARI outpatient visits using data from multiple facilities were seen post-PCV13. Further analysis using a larger and more representative database, such as subnational or national data, could better assess the impact of PCV13 on pneumonia in Zimbabwe.

## Supplementary Data

Supplementary materials are available at *Clinical Infectious Diseases* online. Consisting of data provided by the authors to benefit the reader, the posted materials are not copyedited and are the sole responsibility of the authors, so questions or comments should be addressed to the corresponding author.

## Supplementary Material

ciz462_suppl_Supplemental-Data

## Notes

***Financial support.*** This work was partially supported by Gavi, the Vaccine Alliance, through the CDC Foundation (under memorandum of understanding #971-17). Financial support for sentinel site surveillance was provided by Gavi, the Vaccine Alliance, through a grant to the WHO for the African Paediatric Bacterial Meningitis Surveillance Network.

***Supplement sponsorship.*** This supplement was supported with funds from Gavi, the Vaccine Alliance through The World Health Organization and the CDC Foundation, and The Medical Research Council Unit The Gambia at the London School of Hygiene and Tropical Medicine.

***Potential conflicts of interest.*** All authors: No reported conflicts of interest. All authors have submitted the ICMJE Form for Disclosure of Potential Conflicts of Interest. Conflicts that the editors consider relevant to the content of the manuscript have been disclosed.
