# Supplementary material for: Pneumococcal Conjugate Vaccine Impact on Meningitis and Pneumonia Among Children Aged <5 Years—Zimbabwe, 2010–2016
Source: Clin Infect Dis. 2019 Sep 5;69(Suppl 2):S72–80. doi: 10.1093/cid/ciz462 (PMC6761317; doi:10.1093/cid/ciz462)
Supplement: ciz462_suppl_Supplemental-Data [file ciz462_suppl_supplemental-data.docx]

**Appendix**

| **ICD9 code—2010-2013** | **Condition name—2014-2016**  **(condition name reference, not specifically used in data tables)** |
| --- | --- |
| 4661 | ACUTE BRONCHIOLITIS |
|  | ACUTE BRONCHIOLITIS DUE TO RESPIRATORY SYNCYTIAL VIRUS |
| 4661 | ACUTE BRONCHIOLITIS, UNSPECIFIED |
| 2639 | UNSPECIFIED PROTEIN-ENERGY MALNUTRITION |
| 262 | UNSPECIFIED SEVERE PROTEIN-ENERGY MALNUTRITION (Other severe protein–calorie malnutrition) |
| 260 | KWASHIORKOR |
| 2630 | MODERATE PROTEIN-ENERGY MALNUTRITION (Malnutrition of moderate degree) |
|  | PROTEIN-ENERGY MALNUTRITION OF MODERATE AND MILD DEGREE |
| 2631 | (Malnutrition of mild degree) |
| 3229 | MENINGITIS, UNSPECIFIED |
| 0479 | VIRAL MENINGITIS |
| 3209 | BACTERIAL MENINGITIS, UNSPECIFIED (Bacterial meningitis due to unspecified bacterium) |
|  | MENINGITIS IN MYCOSES |
| 0479 | VIRAL MENINGITIS, UNSPECIFIED |
| 0478 | (Other specified viral meningitis) |
| 7700 | CONGENITAL PNEUMONIA, UNSPECIFIED |
|  | CONGENITAL PNEUMONIA DUE TO CHLAMYDIA |
| 7700 | CONGENITAL PNEUMONIA |
| 4809 | CONGENITAL PNEUMONIA DUE TO VIRAL AGENT |
| 7700 | CONGENITAL PNEUMONIA DUE TO ESCHERICHIA COLI |
| 485 | BRONCHOPNEUMONIA, UNSPECIFIED |
| 481 | LOBAR PNEUMONIA, UNSPECIFIED |
|  | PNEUMONIA DUE TO OTHER INFECTIOUS ORGANISMS, NOT ELSEWHERE CLASSIFIED |
| 4829 | PNEUMONIA, UNSPECIFIED (Bacterial pneumonia, unspecified) |
| 486/4860 | PNEUMONIA, UNSPECIFIED (Pneumonia, organism unspecified) |
| 483 | Pneumonia due to other specified organism |
| 4809 | VIRAL PNEUMONIA, UNSPECIFIED |
| 4848 | PNEUMONIA IN BACTERIAL DISEASES CLASSIFIED ELSEWHERE (Pneumonia in infectious diseases classified elsewhere) |
| 4808 | OTHER VIRAL PNEUMONIA (Pneumonia due to other virus not elsewhere classified) |
| 4801 | (Pneumonia due to adenovirus) |
| 4871 | Influenza with other respiratory manifestations |
|  | (Pneumonia due to RSV) |
| 480  (Block title for 4800-4809) | Viral pneumonia |
